# The serum levels of FGF23, sclerostin, osteoprotegerin do not explain the inverse relationship between coronary calcifications and bone mineral density evaluated using computed tomography

**DOI:** 10.3389/fcvm.2025.1583124

**Published:** 2025-06-25

**Authors:** Laurence Ferrières, Michel Laroche, Yannick Degboé, Acil Jaafar, Jean Ferrières

**Affiliations:** ^1^ Centre de Rhumatologie, CHU Purpan & Université Toulouse III, Toulouse, France; ^2^ Renal Physiological Exploration Department, CHU Rangueil, Toulouse, France; ^3^ Department of Cardiology, UMR INSERM 1295, Toulouse Rangueil University Hospital, Toulouse, France

**Keywords:** osteoporosis, coronary calcification, FGF-23, osteoprotegerin, sclerostin

## Abstract

**Background:**

Osteoporotic patients are at a higher risk of stroke or myocardial infarction compared to non-osteoporotic patients, and conversely, individuals who have experienced a myocardial infarction or stroke are at increased risk for low bone mineral density (BMD) or osteoporotic fractures. Some studies suggest that the relationship between osteoporosis and vascular calcification may stem from the dysregulation of common factors that are implicated in both bone remodeling and the formation of calcified vascular plaques.

**Objectives:**

Our primary endpoint was to evaluate the correlation between bone mineral density and calcification score. Our secondary endpoint was to analyse the association between potential shared serum biomarkers and the calcification score or bone status.

**Methods:**

We conducted a retrospective study between May and October 2015 in 94 patients who had undergone a thoracic CT scan, to assess their coronary risk by calculating an Agatston score. The scans were re-analysed to obtain volumetric bone mineral densities (vBMD). We measured osteoprotegerin, FGF23 and sclerostin in frozen serums from these patients.

**Results:**

Patients with a calcium score of 0 had a significantly higher vBMD than patients with a calcium score > 0 (187.7 vs. 162.1, *p* 0.03). This relationship persisted after adjusting for age, sex, BMI and sedentarity (*p* 0.036). There was no significant relationship between FGF23, osteoprotegerin, or sclerostin levels and the calcium score or vBMD.

**Conclusion:**

Lower vertebral thoracic bone mineral density is significantly associated with an increased risk of vascular calcification. However, this relationship is not explained by the serum levels of FGF23, sclerostin, or osteoprotegerin.

## 1 Introduction

Idiopathic osteoporosis is a risk factor for long-term mortality, and in particular, it doubles the risk of cardiovascular mortality (1).

Osteoporotic patients are more likely to have a stroke or a myocardial infarction than non-osteoporotic patients, and patients who have had a myocardial infarction or a stroke have an increased risk of low BMD (Bone Mineral Density) or osteoporotic fracture (2–5).

This link appears to exist independently of classic cardiovascular or osteoporosis risk factors (smoking, hypertension, diabetes, family history) (6, 7).

Some studies suggest that the links between osteoporosis and vascular calcification could therefore result from secretion anomalies of identical factors potentially implicated in both bone remodelling and calcified vascular plaque formation (8–12).

The mechanism of osteoporosis involves the release of calcium from bone. However, the idea that this liberated calcium subsequently deposits onto the vascular wall is not plausible. The highly precise regulation of calcium homeostasis—and the typically normal levels of serum calcium and phosphate in idiopathic osteoporosis, despite the massive amount of calcium released from bone—suggests the need for more subtle and likely multifactorial explanations.

One possible hypothesis is that, since bone is a vascularized organ, chronic impairment of intraosseous blood flow—secondary to atherosclerosis—might promote bone demineralization.

Finally, the central hypothesis of our work is the existence of a shared pathophysiological mechanism between osteoporosis and atherosclerosis.

Vascular calcification was long considered a passive, unregulated process. It is now better understood that vascular calcification involves both passive and active mechanisms, including a form of mineralization mediated by cells resembling osteoblasts.

These “osteoblast-like cells” exhibit a phenotype very similar to bone osteoblasts and possess an innate capacity for mineralization. They are formed through the differentiation of certain vascular wall cells.

It is reasonable to suppose that factors regulating bone metabolism may also influence these cells. Among the implicated factors are Fibroblast Growth Factor 23 (FGF23), osteoprotegerin (OPG), and sclerostin.

FGF23 was initially identified in patients with tumor-induced osteomalacia (phosphaturic mesenchymal tumors). It is synthesized by osteocytes and plays a key role in phosphate metabolism: it increases renal tubular phosphate excretion and inhibits the hydroxylation of 25-hydroxyvitamin D into 1,25-dihydroxyvitamin D (calcitriol) (13, 14). FGF23 requires the co-receptor Klotho for its activity; Klotho is expressed in the kidney, choroid plexus, germ cells, and arterial walls, and is essential for FGF23 signaling.

In animal models, Klotho gene deficiency leads to premature aging, reduced life expectancy, and transdifferentiation of endothelial cells into osteoblast-like cells, which initiate calcification and thus contribute to atherosclerosis (15). Conversely, overexpression of Klotho—stimulated by 1,25-dihydroxyvitamin D—results in inhibition of vascular calcification.

In animals, FGF23 activity thus appears to be inversely correlated with the risk of vascular calcification. Surprisingly, in humans, FGF23 seems to have the opposite effect. Patients with chronic kidney disease (CKD) often show reduced Klotho expression. In end-stage renal disease (ESRD) patients undergoing hemodialysis, hyperphosphatemia leads to elevated serum FGF23 levels as a compensatory response to increase phosphate excretion.

The combination of high FGF23 and phosphate levels with low Klotho expression is associated with increased cardiovascular mortality (16) and vascular calcification. However, it remains unclear whether this effect is due to the direct vascular toxicity of phosphate—suspected to induce the transformation of vascular smooth muscle cells into osteoblast-like cells (13)—or to dysfunction of the FGF23/Klotho axis.

Osteoprotegerin (OPG) is involved in the regulation of bone resorption. It is a soluble receptor secreted by various cell types, notably osteoblasts.

OPG functions as a decoy receptor by binding to RANK Ligand (Receptor Activator of Nuclear Factor κB Ligand), which would otherwise activate osteoclastogenesis via the RANK receptor. Initially considered a bone-specific protein, OPG was later identified in atherosclerotic plaques. The “osteoblast-like cells” present in these plaques are therefore likely regulated by the same factors as bone osteoblasts, including the RANK–RANK Ligand–Osteoprotegerin pathway. In animal models, OPG deficiency is associated with vascular calcification and increased incidence of fragility fractures (17).

Conversely, in humans, elevated OPG levels have been associated with higher coronary artery calcium scores (18) as well as with the presence and severity of coronary artery disease (19).

Sclerostin is a protein primarily produced by osteocytes that inhibits the WNT signaling pathway, which is involved in the differentiation of osteoprogenitor cells into osteoblasts.

From a cardiovascular perspective, sclerostin may serve as a marker of cardiovascular mortality in hemodialysis patients (20), and has been identified as a risk factor for carotid (21) and aortic calcification (22).

It can be hypothesized that, much like in bone, sclerostin acts as a protective factor against calcification in the vascular wall. The slower progression of vascular calcification may explain the observed survival benefits in patients with higher circulating levels of sclerostin.

The main objective of this work was to identify an inverse correlation between bone mineral density and coronary calcification score using the same CT-scan.

The secondary objectives were to identify the association of FGF23, sclerostin and osteoprotegerin serum levels and the calcification score and bone status.

## 2 Methods

### 2.1 Study population

Our study was conducted in a BIOCAC population (Biological phosphocalcic metabolism and coronary artery calcifications, RCB ID Number: 2015-A00853-46) (Appendix 1).

The BIOCAC protocol was an observational, monocentric study. The study population included patients from the Centre for Detection and Prevention of Atherosclerosis at Toulouse University Hospital, recruited between May and October 2015. As part of this protocol, these subjects had a chest CT scan to calculate a coronary calcification score.

Patients with the following characteristics were initially included in the BIOCAC protocol: over 18 years of age, in primary prevention for coronary pathology (with no history of acute coronary syndrome, and no documented coronary stenosis greater than 50%), with an intermediate cardiovascular risk based on the European SCORE (Systematic Coronary Risk evaluation) equation (risk of cardiovascular death at 10 years greater than or equal to 1% and less than 5%), having signed the informed consent and affiliated to a national insurance.

Patients with the following characteristics were excluded because of potential biases: chronic renal insufficiency with an estimated GFR of less than 60 ml/min/1.73 m^2^ (due to changes in their phosphocalcic metabolism), receiving diabetes treatment, capillary glucose greater than or equal to 1.10 g/L confirmed by a plasma glucose level greater than or equal to 1.10 g/L, receiving diuretic treatment (due to a modification of the urinary ionogram), seropositive for HIV, on antiretrovirals (due to a modification of the urinary ionogram), pregnant women, and patients under the protection of justice, guardianship, or curatorship.

We included 94 patients from this population, corresponding to those who had undergone all the exams under the initial protocol and for whom serum was available for complementary biological assays. The sample size of 94 participants was determined based on the availability of eligible patients within the study period who met all inclusion criteria and had complete biomarker, vBMD, and CAC data. While a formal *a priori* power calculation was not conducted due to the exploratory nature of the study, the sample size was considered sufficient to detect moderate to large effect sizes in the primary analyses.

This protocol has been ethically approved by the Limoges CPP Ethics committee.

### 2.2 Data collection

We collected the information on the patients' demography (sex, age, weight and height), cardiovascular risk factors, and osteoporosis risk factors from the BIOCAC database.

Additional data, not available in the BIOCAC data collection, was collected either from the medical files or by direct contact with patients.

#### 2.2.1 Coronary artery calcium score

Coronary calcifications were evaluated by calculating an Agatston calcification score. This was determined with a non-injected cardiac scanner. A Siemens device (Definition 64 strips) was used. The average irradiation was about 1 mSv 49.

A calcification score strictly lower than 1 corresponds to a null cardiovascular risk, a score greater than 100 predicts a high risk of events in 2–5 years, with an annual risk greater than 2% (23–26).

#### 2.2.2 Bone mineral density

In our cohort, the CT scans performed to assess coronary calcifications were re-analysed to determine the volumetric bone mineral density (vBMD) on the 5th thoracic vertebra, as described in the study by Schreiber et al. (27).

This vertebra was chosen because it gave a better image quality compared to other vertebras on our CT scans.

**Figure 1:**
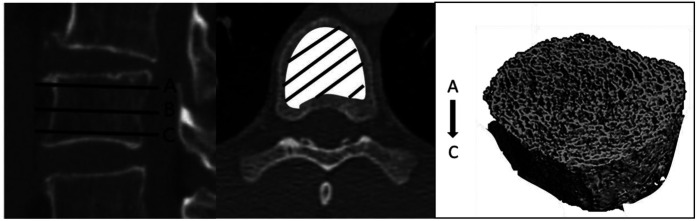
Measurement of volumetric bone density.

The vertebra of interest was first analysed on a sagittal section, to include the entire vertebral body on the vertical axis. Benchmarks were taken on the upper (A), middle (B) and lower (C) vertebrae. The vertebra was then worked on an axial section, to include the entire body of the vertebra on the horizontal axis at each level of the vertebra (A: upper, B: median, C: lower). A volume in 3 dimensions (from A to C) corresponding to the vertebral body (Figure 1) was then built.

Finally, this volume was studied to obtain the average volumetric bone density ​in Hounsfield units. These measurements were blindly performed by two independent observers. Disagreement was solved by consensus. The coefficient of variation of this measurement, intra- and inter-observer, was established for 20 consecutive patients.

#### 2.2.3 Biological measurements

We performed additional assays, including FGF23, sclerostin, and osteoprotegerin, using serum initially collected under the BIOCAC protocol (Eurobio Scientific: ELISA kits with final fluorometric detection, Kit Elisa Bone Alkphos Human Marker Ce, Kit Elisa Sclerostin High Sensitive Human Marker Ce, Kit Elisa Osteoprotegerin Human Marker Ce).

### 2.3 Statistical analyses

The mean vBMD and the coronary artery calcium SC were first compared using Spearman's correlation. The patients were then divided into two groups: those with a coronary artery calcium score of zero (cardiovascular risk zero) and those with a coronary artery calcium score strictly higher than zero (cardiovascular risk present). We assessed the distribution of vBMD by coronary artery calcium score using the chi-squared test, and the population characteristics using Fisher's exact test.

We made an adjustment using two different models to confirm our working hypothesis (linear regression model and multinomial logistic regression model). We employed two regression models to demonstrate the robustness and coherence of our results. Both models were designed to account for the key confounding factors identified in the study, including age, sex, BMI, and sedentary lifestyle. By adjusting for these variables, we aimed to minimize potential bias and better isolate the associations of interest.

We evaluated the relationships between the biological markers (FGF 23, Ostopotegerin, Sclerostin), the mean vertebral vBMD, and the calcium score with Student's *t*-test.

## 3 Results

### 3.1 Description of the study population

Ninety-four patients were included, with an average age of 64.6 years, 47 women and 47 men. Eleven (13.1%) patients had a known osteoporosis. Four (4.8%) patients had a history of osteoporotic fracture (Table 1).

**Table 1:** Description of the study population.

Study population	*N* = 94
Demographic data	
Mean age (min-max)	64.6 (28–86)
Sex (males, %)	47 (50)
Mean BMI, kg/m^2^ (min-max)	25.4 (17.5–43.1)
Osteoporosis risk factors (%)	
Age > 60 years	73 (77.7)
History of osteoporotic fracture	4 (4.8)
Amenorrhoea	0 (0)
Early menopause	0 (0)
Osteoporosis-inducing chronic disease	7 (8.3)
Extended immobilization	0 (0)
Corticosteroids	1 (1.2)
BMI < 19 kg/m^2^	0 (0)
Active smoking	13 (15.5)
Excessive consumption of alcohol	2 (2.4)
Low calcium intake (<800 mg/day)	14 (16.7)
Vitamin D	
Deficiency (Vitamin D < 10 ng/ml)	7 (8.3)
Insufficiency (Vitamin D = 10–30 ng/ml)	51 (60.7)
Sedentarity	6 (7.1)
Cardiovascular risk factors (%)	
Family history of cardiovascular disease	25 (29.8)
Familial dyslipidaemia and hypercholesterolaemia	83 (98.8)
Diabetes	4 (4.8)
Hypertension	32 (34.0)
Coronary artery calcium score	
Mean	272.7
Median	8
Standard deviation	692.2
Minimum-Maximum	0–4,717
Coronary artery calcium score, distribution by risk class (%)	
0	34 (36.2%)
>0	60 (63.8%)
vBMD (HU)	
Mean	171.3
Median	163
Minimum-Maximum	87.2–309
Standard deviation	52.6

Number (percentage) or mean (standard deviation).

The main risk factor for osteoporosis was age (over 60 years: 77.7%). Thirteen patients (15.5%) were active smokers, 14 (16.7%) had low calcium intake, 7 (8.3%) had a profound vitamin D deficiency, 2 (2.4%) had excessive alcohol consumption and 6 (7.1%) were sedentary. Finally, 7 (8.3%) patients had an osteoporosis-inducing chronic disease (one hyperparathyroidism, four diabetics, one spondyloarthropathy, one Crohn's disease).

Regarding cardiovascular risk factors, 83 (98.8%) patients had familial dyslipidaemia or hypercholesterolaemia, 25 (29.8%) had hereditary cardiovascular disease, and 4 (4.8%) had diabetes.

### 3.2 Primary endpoint

Thirty-four patients had a coronary artery calcium score of 0 (36.2%) and 60 patients had a score strictly higher than 0 (63.8%). As shown in Table 2, the patients with a coronary artery calcium score of 0 had a significantly higher vBMD (187.7 vs. 162.1, *p* 0.03).

**Table 2:** vBMD and coronary artery calcium score.

Coronary artery calcium score by mean vBMD	Coronary artery calcium score = 0	Coronary artery calcium score > 0	*p* *
	*n* = 34 (36.2%)	*n* = 60 (63.8%)	
Mean vBMD (HU)	187.7 (59.6)	162.1 (46.2)	*p* = 0.03

Mean (standard deviation).

* Fisher's exact test.

A comparison of the characteristics of these two groups showed a significant difference between each group for age, sex, BMI and physical activity (see Table 3).

**Table 3:** Comparison of the characteristics of the two groups.

Coronary artery calcium score by population characteristics	Coronary artery calcium score = 0	Coronary artery calcium score > 0	*p*
	*n* = 34 (%)	*n* = 60 (%)	
Mean vBMD (HU)	187.7 (59.6)	162.1 (46.2)	*p* = 0.03
Demographic data			
Mean age (min-max)	60.1 (11.8)	67.2 (9.0)	0.002
Sex (males, %)	35.3	58.3	0.032
Mean BMI, kg/m^2^ (min-max)	26.7 (5.5)	24.7 (31)	0.03
Osteoporosis risk factors (%)	11.8	11.7	0.99*
Age > 60 years	35.3	15.0	0.023
History of osteoporotic fracture	0	6.7	0.29*
Amenorrhoea	0	0	
Early menopause	0	0	
Osteoporosis-inducing chronic disease	4.4	2.2	0.99*
Extended immobilization	0	0	
Corticosteroids	0	1.7	0.99*
BMI < 19 kg/m^2^	0	3.3	0.53*
Active smoking	20.6	10.0	0.14*
Excessive consumption of alcohol	0	3.3	0.75*
Low calcium intake (<800 mg/day)	14.7	15.0	0.97
Vitamin D			
Deficiency (Vitamin D < 10 ng/ml)	11.3	5.5	0.24*
Insufficiency (Vitamin D = 10–30 ng/ml)	60.0	60.0	1
Sedentarity	14.7	1.7	0.03*
Cardiovascular risk factors (%)			
Family history	20.6	30.0	0.32
Familial dyslipidaemia and hypercholesterolaemia	70.6	86.7	0.06
Diabetes	8.8	6.7	0.71*
Hypertension	0	5.0	0.55*
Cardiovascular risk factors (%)	12	20	0.44*

Number (percentage) or mean (standard deviation).

* Fisher's exact test.

The “zero coronary artery calcium score” group had significantly more patients over 60 (35.3% vs. 15%, *p* 0.023), there were fewer men (35.3% vs. 67.2%, *p* 0.032), they were more sedentary (14% vs. 1.7%, *p* 0.03) and had a higher mean BMI (26.7 vs. 24.7 kg/m^2^, *p* 0.03).

The total distribution of risk factors for osteoporosis was not significantly different between the 2 groups (11.8% vs. 11.7%, *p* 0.99). The coefficient of variation for the intra-observer vBMD measurement was 1.2%, and the coefficient of variation for the inter-observer measurement was 3.6%.

Since these factors are potentially confounding factors, we made an adjustment using two different models to confirm our working hypothesis.

We first used a linear regression model: the relationship between vBMD and coronary artery calcium score persisted after adjusting for age, sex, BMI and sedentary lifestyle (*p* 0.036). We then used a multinomial logistic regression model: the relationship between coronary artery calcium score and vBMD remained significant (*p* 0.017).

### 3.3 Secondary endpoints

The FGF23, osteoprotegerin and sclerostin values did not differ for the subjects with an coronary artery calcification score < 0 compared to those > 0 (Table 4).

**Table 4:** Biomarkers according to the calcium score.

Biological measurements	Coronary artery calcium score		
	0	>0	*p* *
FGF 23 (10 à 50 pg/ml)	49.9 (13.6)	48.6 (15.9)	0.71
Sclerostin (0.68–5.45 ng/ml)	0.703 (0.21)	0.720 (0.23)	0.74
Osteoprotegerin (0.01–5.32 pmol/L)	4.211 (1.20)	4.379 (1.15)	0.82

Mean (standard deviation).

* Student's *t*-test comparison of the biomarkers according to coronary artery calcium score.

FGF23 was not significantly related to calcium score (*p* = 0.14) nor to mean vBMD (*p* = 0.76). This absence of a significant relationship persisted after separating the calcium scores into classes (*p* = 0.71).

Sclerostin levels were not significantly associated with calcium score (*p* = 0.88) or mean vBMD (*p* = 0.53). This lack of significant relationship persisted even after stratifying calcium scores into classes (*p* = 0.74). Similarly, osteoprotegerin was not significantly associated with calcium score (*p* = 0.74) or mean vBMD (*p* = 0.26), and this finding remained unchanged when calcium scores were categorized (*p* = 0.82).

## 4 Discussion

A total of 94 patients (47 women and 47 men) were included, with a mean age of 64.6 years. Among them, 34 patients had a coronary artery calcium (CAC) score of 0 (36.2%), while 60 patients had a CAC score greater than 0 (63.8%). The mean vBMD was 171.3 HU. Patients with a zero CAC score, indicating low cardiovascular risk, had significantly higher vBMD compared to those with a high calcium score (187.7 vs. 162.1 HU, *p* = 0.03). This relationship between BMD and calcium score persisted after adjusting for potential confounders (age, sex, BMI, and sedentary lifestyle), suggesting that the impact of these factors on the observed relationship is minimal. Thus, higher cardiovascular risk was significantly associated with lower bone mineral density.

### 4.1 Reliability of the vBMD measurement

The mean vBMD in our study was 171.3 HU, using T5 vertebra. Patients with a zero coronary artery calcium score had a mean vBMD of 187.7 HU and those with a coronary artery calcium score higher than zero had a mean vBMD of 162.1 HU; the delta between our two groups was 25.6 HU.

Most of the studies using computed tomography to evaluate bone status concerned the lumbar spine. On DXA measurements at L1–L4, Schreiber et al. (27) found mean values in 25 subjects of 133.0 HU (95% CI [118.4–147.5]), 100.8 HU (95% CI [93.1–108.8]), and 78.5 UH (95% CI [61.9–95.1]) for normal, osteopenic and osteoporotic patients respectively. Hendrickson et al. (28) meanwhile found mean values in 252 patients of 153 and 129.6 HU for osteopenic and osteoporotic patients. Lee et al. (29) established mean L1 values in 571 patients of 122.1 HU for normal patients and ≤110 HU for osteoporotic patients (DXA). Finally, at the thoracic level (T6), Marinova et al. (30) found mean values in 234 patients of 160–200 HU in normal patients, 130–160 HU in osteopenic patients and 60–130 HU in osteoporotic patients. These values seem comparable to ours.

The reproducibility of the CT measurements in these studies is 1%–3% (31, 32), comparable to our coefficients of variation of 1.2% and 3.8%.

Other studies have confirmed a close correlation between bone mineral density measured by DXA and three-dimensional bone mineral density assessed by CT scan (33–37).

### 4.2 The relationship between bone density and vascular calcification in the literature

Numerous studies have demonstrated an inverse relationship between bone mineral density and vascular calcification:

The Framingham study (38) aimed to establish the relationship between volumetric vertebral bone mineral density and coronary, aortic and valvular calcifications. It showed that coronary calcification was inversely related to bone density in women but not in men, and that aortic calcification was inversely related to bone mineral density in both men and women. They found no significant relationship for valvular calcifications. Additionally, the Multi-Ethnic Study of Atherosclerosis (MESA) (39), aiming to evaluate the association between lumbar vertebral bone mineral density and coronary and aortic calcifications, showed an inverse relationship between coronary calcifications and bone mineral density in men and women. Two other studies, the SWAN study (Study of Women's Health Across the Nation) (40) and Schulz et al.'s (41) study (conducted only in women), also showed that women with low vertebral bone mineral density had a higher risk of aortic (but not coronary) calcification.

These results are also true for density measurements from other bone sites.

The Rancho Bernardo Study (42) found a significant association between femoral bone mineral density and coronary calcification, and Jørgensen et al. (43) identified the same relationship for wrist density and carotid calcifications. A study by Kiel et al. (44) showed in women, but not in men, that bone loss in the metacarpal bone (measured by hand radiographs) was associated with aortic calcification. Hak et al. (15) and Boukhris et al. (45) found an identical relationship in postmenopausal women. A study by Chen et al. (46) found a significant correlation in women, but not in men, between low total bone mineral density and high coronary artery calcium score (>100).

The HUNT Study (47), a Norwegian cohort study involving 22,857 adults, found no significant association between distal forearm bone mineral density (BMD) and the risk of cardiovascular diseases, including atrial fibrillation, acute myocardial infarction, ischemic stroke, hemorrhagic stroke, and heart failure.

The CKD Study (48), which included 1,957 patients with predialysis chronic kidney disease, indicated that lower BMD was associated with an increased risk of major adverse cardiovascular events and accelerated progression of coronary artery calcification.

The OUP Academic Asian Women Study (49), involving 12,681 women aged 50–80, revealed that lower BMD at the lumbar spine, femoral neck, and total hip was independently associated with a higher risk of atherosclerotic cardiovascular events.

Finally, a meta-analysis (50) analyzing data from 46,182 participants concluded that lower BMD is associated with an increased risk of all-cause and cardiovascular mortality, although no significant link was found with stroke mortality.

The discrepancies in the results of these different studies can be explained by differences between the populations, methodology and imaging modalities. Some results appear to find differences depending on gender which may indicate differences in the physiopathology of vascular calcification and bone demineralization between men and women. In women, rapid trabecular bone loss at the onset of menopause is paralleled by increased development of atherosclerosis in the aorta (51–53). Further gender analyses should be done to obtain consistent results. However, these findings were not modified after adjustment for cardiovascular and osteoporosis risk factors (except for age, which in some of these studies may partially mitigate the relationship between bone density and vascular calcification).

### 4.3 What could the physio-pathological links between these two diseases be?

Our study is the first to establish a link between coronary calcifications and bone mineral density, both measured using a single chest computed tomography scan, while also exploring the underlying mechanisms. For our cohort, we analyzed three biological parameters that, based on existing literature, we identified as the most relevant potential candidates to explain the relationship between these two conditions.

Indeed, the cells recruited on the lipid plaques in atherosclerosis, known as “osteoblast-like calcifying vascular cells”, have phenotypes very close to the cells involved in bone remodelling. Proteins initially considered to be characteristic of bone tissue have been identified in these atheroma plaques: BMP2, osteopontin, Gla protein, osteoprotegerin. These cells are probably subjected to the same regulatory factors, such as Rank-RankL-Osteoprotegerin or Wnt protein-Beta catenin pathways (8, 54–56).

Osteoprotegerin (OPG) is the natural inhibitor of the Rank Rank-Ligand pathway and therefore a potent inhibitor of bone resorption. Mice deficient in osteoprotegerin have been shown to develop vascular calcifications and bone fragility with fracture. In rats, the injection of OPG prevents vascular calcifications caused by Warfarin. In haemodialysis subjects, OPG levels are associated with the coronary calcification score. In humans, elevated serum OPG levels are associated with coronary artery disease and cardiovascular mortality. Indeed, following a myocardial infarction, the OPG level predicts the risk of subsequent mortality. OPG knock-out mice were shown to develop osteoporosis (17), and in postmenopausal women there is a connection between OPG gene polymorphisms and bone mineral density (57, 58).

Sclerostin is a physiological inhibitor of the Wnt pathway, known to stimulate osteoblastic activity. It is believed to be a marker of cardiovascular mortality in dialysis patients (59). In another study, sclerostin was negatively correlated with bone mineral density in men and women without renal failure and with the size of the calcified vascular plaques (60).

FGF23, initially discovered in oncogenic phosphorus diabetes, is known to be involved in the regulation of phosphoraemia and mineralisation (61, 62). In patients with renal failure, increased levels of FGF23 and phosphorus and decreased levels of Khlotho (FGF23 co-receptor) are connected with increased mortality from cardiovascular disease (63). Several studies have shown that there is a relationship between FGF23 levels and cardiovascular mortality, excluding renal failure (64). The FGF23 level could also be linked to osteoporosis. Mirza et al. have shown that the level of FGF23 was directly related to the risk of fracture in 2,868 Swedish men aged 75.4 ± 3.2 years. Men with a FGF23 level of >55.7 pg/ml had a vertebral fracture risk of 2.30 (1.16–4.58). This relationship persisted after adjustment for BMI, bone mineral density, GFR, and 25(OH)D and PTH levels (65). Lane et al. showed an association between FGF23 levels and risk of fracture in elderly men (13).

A Mendelian randomization study (66) indicated that higher genetically predicted FGF23 concentrations were associated with increased gynoid (pelvic girdle) bone mass but not with lumbar vertebrae or femoral neck bone mineral density (BMD). However, these associations were not significant after excluding certain single nucleotide polymorphisms (SNPs) linked to vitamin D metabolism.

Mendelian randomization studies (67) have also explored the effects of genetically lowered sclerostin on cardiovascular outcomes. The findings are mixed, with some studies suggesting an increased risk of myocardial infarction and hypertension, while others report no significant associations.

Research on men with heart failure (68) revealed significantly higher levels of osteoprotegerin, while sclerostin levels were significantly lower compared to controls. These findings suggest alterations in bone-related proteins in heart failure patients.

Despite the positive results from these studies showing correlations between FGF23 levels and either cardiovascular disease or osteoporosis independently, our study did not observe any significant correlations in the same cohort between FGF23, sclerostin, osteoprotegerin, coronary calcification score, and bone mineral density.

Other pathophysiological hypotheses should be explored, for example, pro-inflammatory cytokines involved in the genesis of atheromatous plaques and in bone resorption (56, 69), the role of oxidative stress and lipids (oxidative stress increases with age and fosters the appearance of oxidised lipids capable of inducing the differentiation of osteoclasts whilst inhibiting that of osteoblasts within bone tissue, and simultaneously promoting their differentiation within the atheromatous plaque (70), or the direct role of ischaemia on bone tissue: we have shown that the intraosseous arterioles could be the site of atherosclerotic lesions and that this atherosclerosis was more serious and more frequently encountered in subjects operated for an osteoporotic fracture of the upper extremity of the femur than for paired subjects having had arthroplasty for coxarthrosis (71). In subjects with asymmetric arteriopathy of the lower limbs, we have shown that the limb, the site of the arterial disease, was demineralized compared to the contralateral limb (72).

The lack of significant relationships between our biomarkers, coronary artery calcifications (CAC), and bone mineral density (vBMD) could be attributed to several factors. These include: timing of biomarker measurement, which may not reflect long-term physiological status relevant to vBMD or calcification processes; differences in study population characteristics (e.g., age range, comorbidities, baseline risk profiles) compared to prior studies (73–76); potential threshold effects or non-linear relationships not captured by the models used; influence of unmeasured confounders or effect modifiers that may dilute observable associations in this specific cohort; tissue-specific regulation, where systemic biomarker levels may not accurately reflect local activity in bone or vascular tissue, and limited statistical power, which may have prevented detection of modest but meaningful associations.

### 4.4 Limitations

The main limitation of this study is its monocentric design. Another limitation is the measurement of vBMD at a single vertebral level, specifically in the thoracic region, using CT scanning. This approach is not commonly used in current clinical practice and is mostly found in recent studies. As of now, there are no established guidelines for interpreting this type of measurement.

Concerning the biomarkers, in our study, we focused exclusively on factors directly related to cardiovascular and bone health, as these were the primary areas of interest. Consequently, other potential confounders, such as iron status, were not measured or available in our study.

## 5 Conclusion

Our study demonstrates that the coronary artery calcium score—and thus cardiovascular risk—is significantly associated with decreased vertebral bone mineral density. Given that this relationship has been previously reported in the literature, it is reasonable to consider that the association may have a pathophysiological basis.

However, taking into account the limitations of our study, we did not find a significant correlation between serum levels of FGF23, osteoprotegerin or sclerostin and either the calcium score or bone mineral density.

This study also highlights the reliability of CT scans in assessing bone status. Therefore, as CT imaging is often performed for various clinical indications, it could also serve as a valuable tool for the systematic and early screening of osteoporosis.

## Data Availability

The original contributions presented in the study are included in the article/Supplementary Material, further inquiries can be directed to the corresponding author.
