# Supplementary material for: The serum levels of FGF23, sclerostin, osteoprotegerin do not explain the inverse relationship between coronary calcifications and bone mineral density evaluated using computed tomography
Source: Front Cardiovasc Med. 2025 Jun 25;12:1583124. doi: 10.3389/fcvm.2025.1583124 (PMC12237962; doi:10.3389/fcvm.2025.1583124)

Supplementary Figure 1a. Histograms by transformation
DMO_MOY

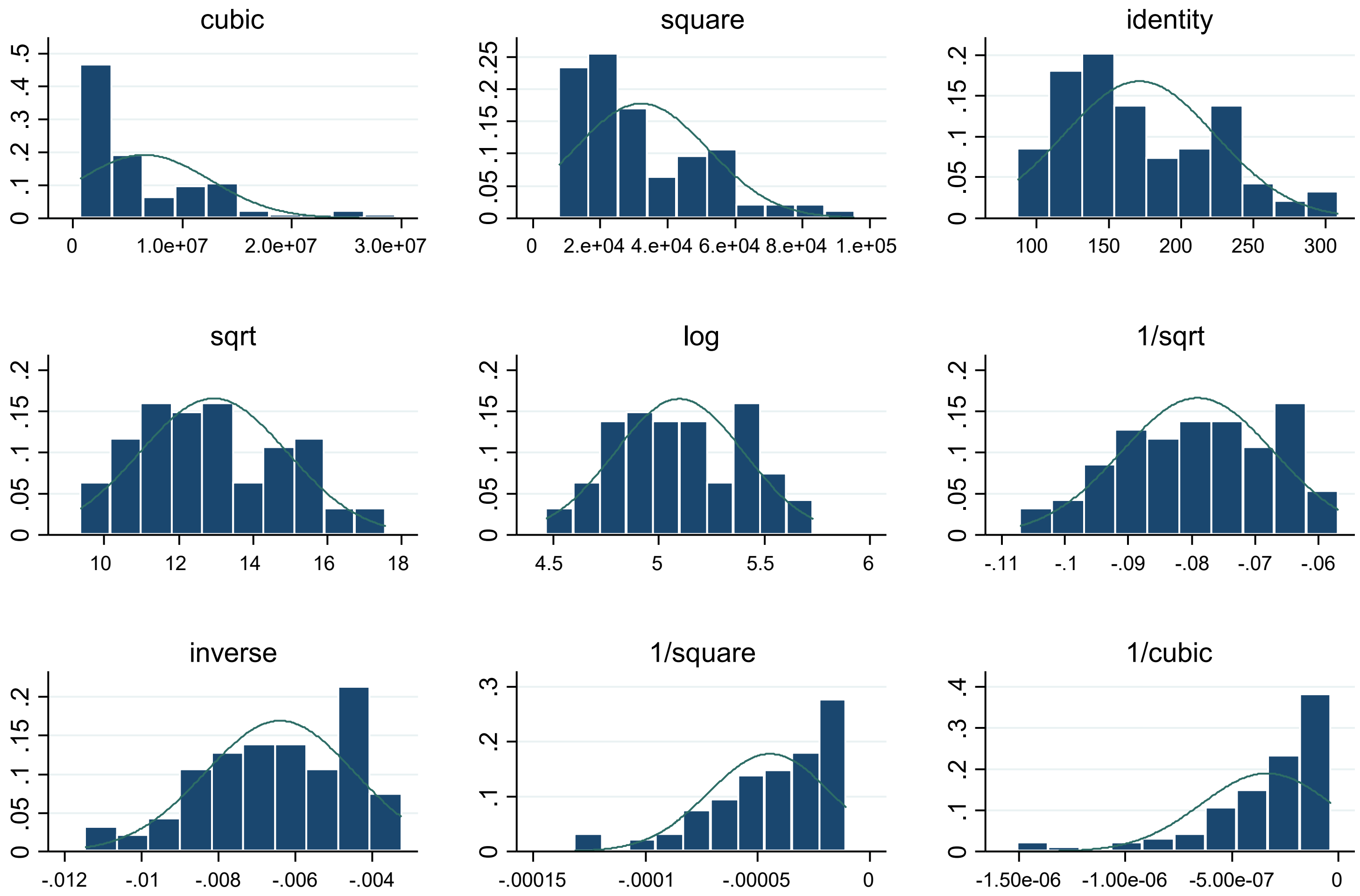

Supplementary Figure 1b. Histograms by transformation
Ca Score

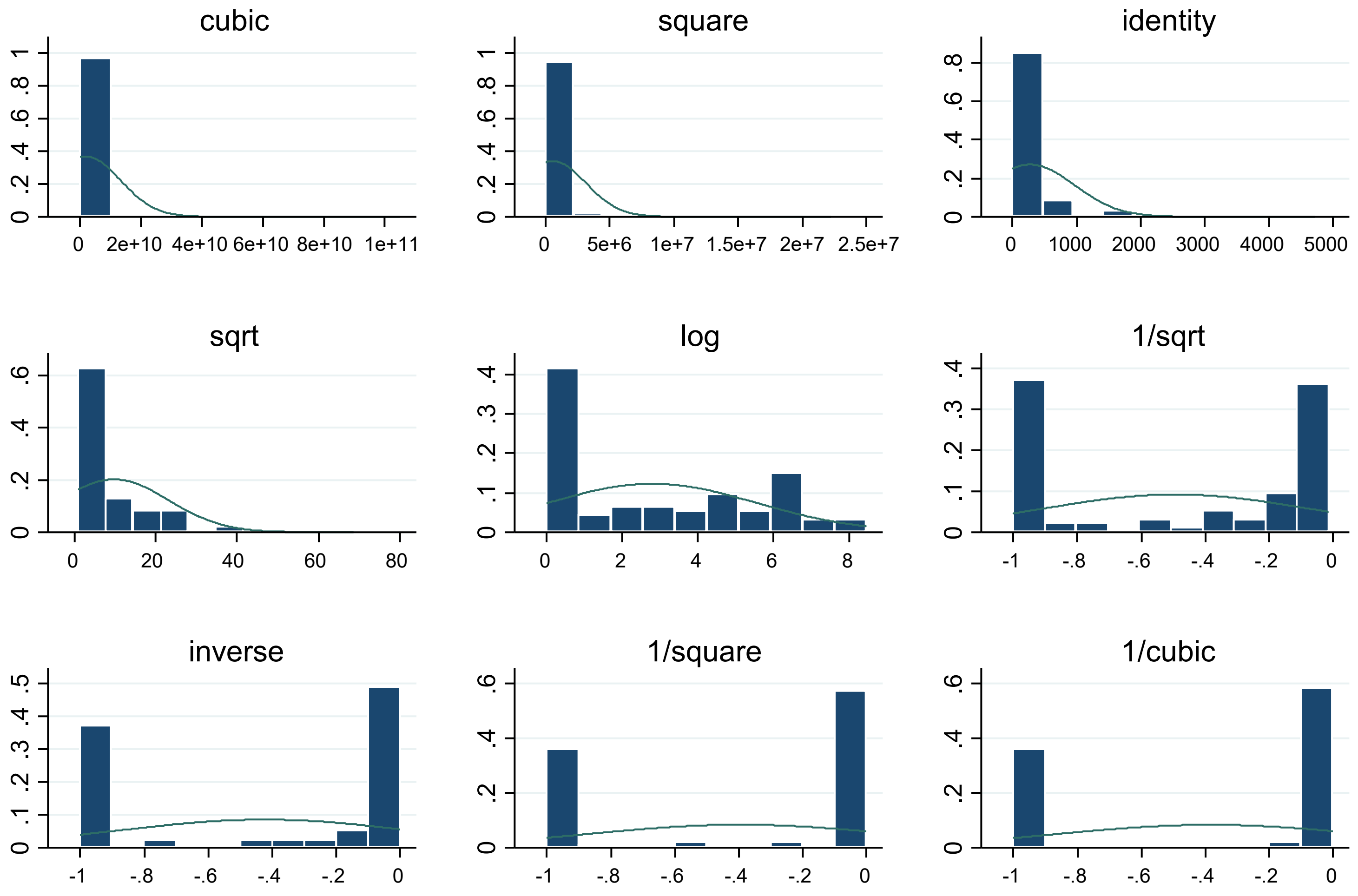

Supplementary Figure 1c. Histograms by transformation
FgF23

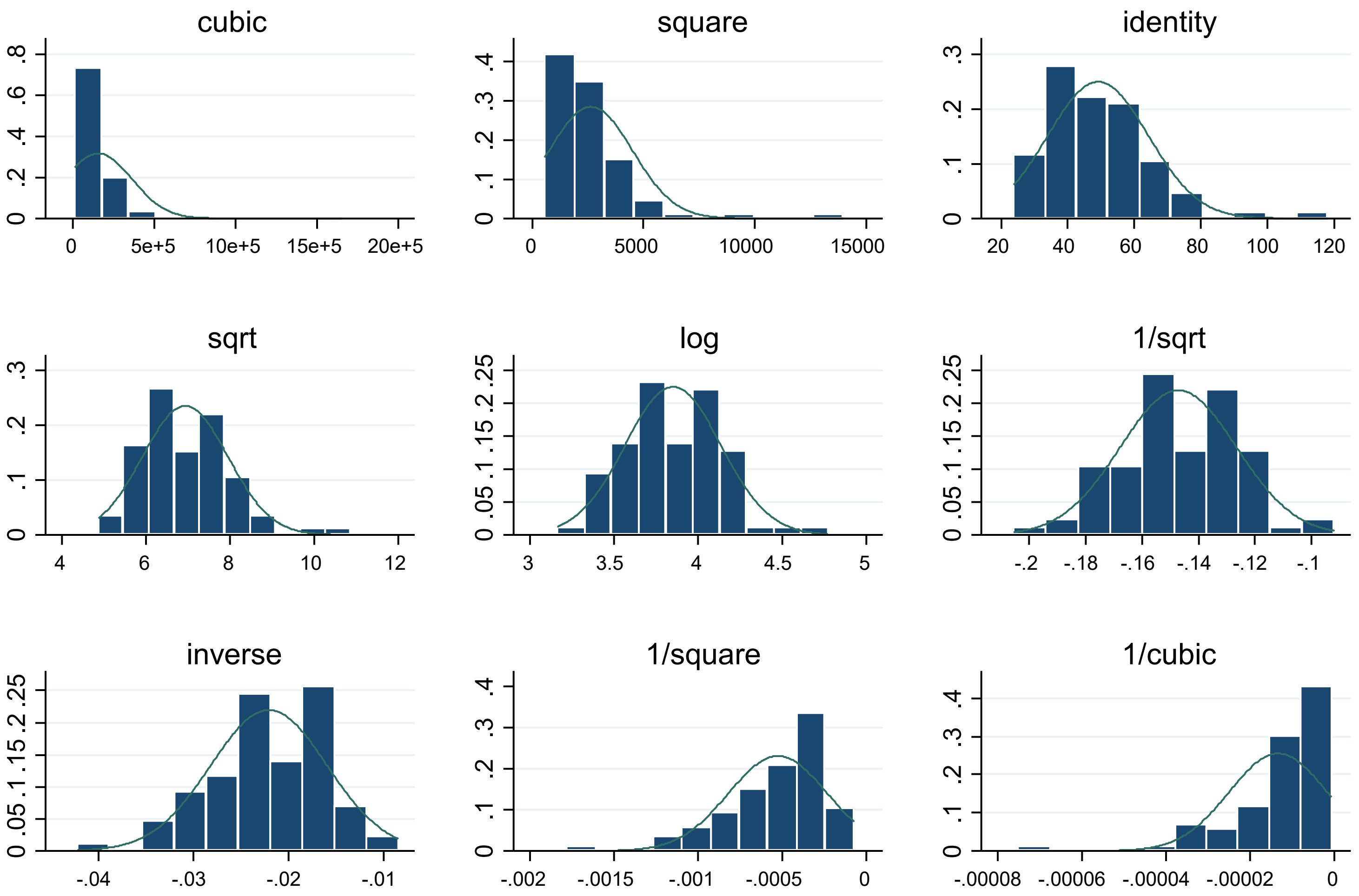

Supplement: Supplementary file 11 [file Image1.pdf]
