# Supplementary figures and images for: The serum levels of FGF23, sclerostin, osteoprotegerin do not explain the inverse relationship between coronary calcifications and bone mineral density evaluated using computed tomography
Source: Front Cardiovasc Med. 2025 Jun 25;12:1583124. doi: 10.3389/fcvm.2025.1583124 (PMC12237962; doi:10.3389/fcvm.2025.1583124)

Supplementary Figure 2. Fit plot of DMO by Ca score  
Quadratic fit function

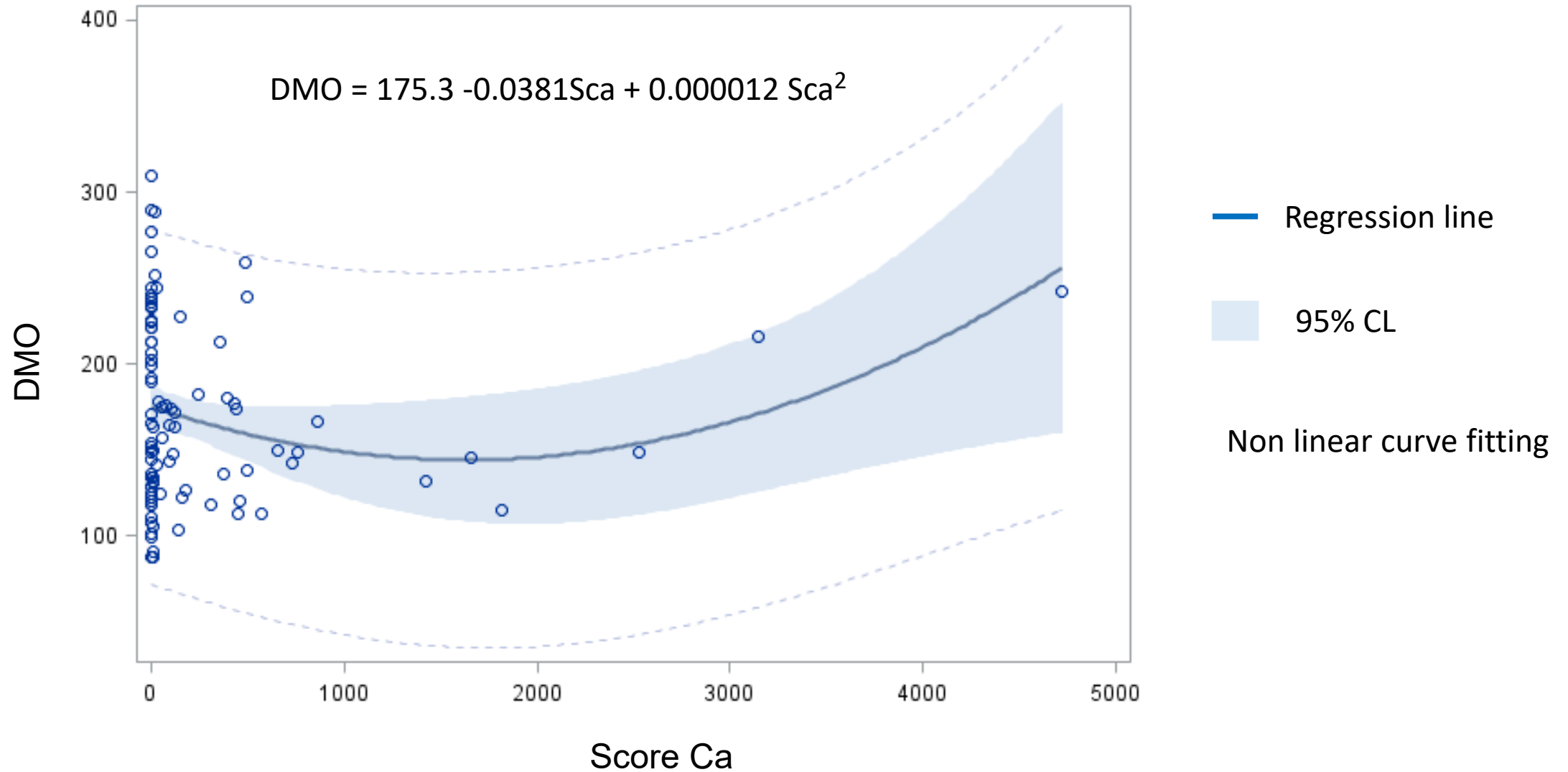

Supplement: Supplementary file 12 [file Image2.pdf]
